# A Novel Asymmetric Trench SiC Metal–Oxide–Semiconductor Field-Effect Transistor with a Poly-Si/SiC Heterojunction Diode for Optimizing Reverse Conduction Performance

**DOI:** 10.3390/mi15040461

**Published:** 2024-03-29

**Authors:** Yiren Yu, Zijun Cheng, Yi Hu, Ruiyi Lv, Shengdong Hu

**Affiliations:** School of Microelectronics and Communication Engineering, Chongqing University, Chongqing 400030, China

**Keywords:** silicon carbide, heterojunction, asymmetric trench MOSFET, low cut-in voltage, switching loss

## Abstract

In this paper, a novel asymmetric trench SiC MOSFET with a Poly-Si/SiC heterojunction diode (HJD-ATMOS) is designed to improve its reverse conduction characteristics and switching performance. This structure features an integrated heterojunction diode, which improves body diode characteristics without affecting device static characteristics. The heterojunction diode acts as a freewheeling diode during reverse conduction, reducing the cut-in voltage (*V*_cut-in_) to a lower level than conventional asymmetric trench SiC MOSFET (C-ATMOS), while maintaining a similar breakdown voltage. Meanwhile, the split gate structure reduces gate-to-drain charge (*Q*_gd_). Through TCAD simulation, the HJD-ATMOS decreases *V*_cut-in_ by 53.04% compared to the C-ATMOS. Both *Q*_gd_ and switching loss are reduced, with a decrease of 31.91% in *Q*_gd_ and 40.29% in switching loss.

## 1. Introduction

The wide bandgap semiconductor properties of silicon carbide (SiC) make it a promising candidate for the development of future power switching devices [1,2]. This is primarily due to SiC possessing properties such as a strong breakdown field, high physical and chemical stability, high thermal conductivity, and high electron saturation velocity [3,4,5]. SiC devices can operate in harsh environments due to their wide band gap of 3.25 eV and high thermal conductivity of 5 W/(cm·K) [6]. The SiC MOSFET is the most significant SiC power switching device due to its lack of trail current. This reduces switching loss and radiator volume, improving system power density [7].

SiC MOSFETs commonly make use of parasitic body-PN diodes as freewheeling diodes (FWD) in power inverter and converter systems [8]. However, parasitic body-PN diodes in SiC MOSFETs are not ideal for use as freewheeling diodes [9]. The reasons for this are as follows: Stacking faults (SFs) in SiC devices may cause reliability issues and increase conduction loss [10]. Although recent papers concerning the measured degradation of SiC MOSFETs [11] show a high level of current threshold (about 5× the nominal current or more than 1000 A/cm^2^) for the starting of bipolar degradation, bipolar degradation effects can still occur in SiC MOSFETs under large cyclic pulse current densities. This will limit the application of SiC MOSFET devices in key areas, such as the surge current that flows through a diode during the start-up of a power converter, which can be more than ten times its rated current [12]. Furthermore, the body diode’s *V*_cut-in_ voltage (~2.7 V) is much higher than that of its silicon counterparts due to SiC’s wide bandgap [13]. To overcome the drawbacks of parasitic body-PN diodes, numerous approaches have been devised to deactivate them. One approach is to integrate SiC MOSFETs with Schottky barrier diodes (SBDs) [14,15,16,17]. However, the use of external diodes not only introduces parasitic inductance, limiting switching frequency, but also consumes additional area in the package [18]. And Schottky contacts suffer from a significant increase in reverse leakage current at high temperatures.

Furthermore, SiC MOSFETs with low-barrier and heterojunction diodes are available [19,20]. Heterojunction diodes formed between polysilicon and SiC are attractive. Shenoy and Baliga [21] and Yamagami et al. [22] presented studies on heterojunction diodes using P-Poly-Si and n-6H-SiC, and Poly-Si and 4H-SiC, respectively. Both studies demonstrated low-forward-voltage Schottky-like characteristics. Ni et al. [23] proposed a trench SiC MOSFET integrating polysilicon/SiC HJD, exhibiting excellent freewheeling diode (FWD) performance in both the first and third quadrants. The HJD’s unipolar behavior, similar to that of a Schottky diode, effectively suppresses the turn-on of the problematic body diode, mitigating the aging degradation observed in conventional SiC MOSFETs. Additionally, HJDs reduce reverse recovery voltage and losses, enhancing long-term operational reliability. Furthermore, HJD integration eliminates the need for a separate SBD, leading to a smaller chip area, simpler packaging, and reduced overall system cost. This also minimizes parasitic inductance arising from additional components.

A novel asymmetric trench SiC MOSFET with a heterojunction diode at the right of the gate trench is proposed and simulated in this paper. The structure includes a trench gate with split-gate electrodes and a thicker P-Poly-Si layer, resulting in reduced gate charge and improved switching performance. To suppress the depletion layer, an n-type doped current spreading layer (N-CSL) is formed under the entire P-well region [24]. To maintain the breakdown voltage (BV) of the device structure while maintaining transfer and output characteristics similar to those of C-ATMOS [25,26,27], the depth of the P-well on the right side is not changed. The N-channel (*N*_ch_) is positioned below the P-Poly-Si and in contact with the CSL. The integrated HJD structure of the proposed device eliminates the requirement for an anti-parallel SiC SBD during reverse conduction. The HJD turns on at a low source–drain voltage (*V*_sd_), thus eliminating bipolar degradation by inactivating the body diode. The split gate results in a decrease in gate charge, leading to a reduction in switching losses in the HJD-ATMOS without affecting other characteristics.

## 2. Device Structure and Mechanism

**Figure 1:**
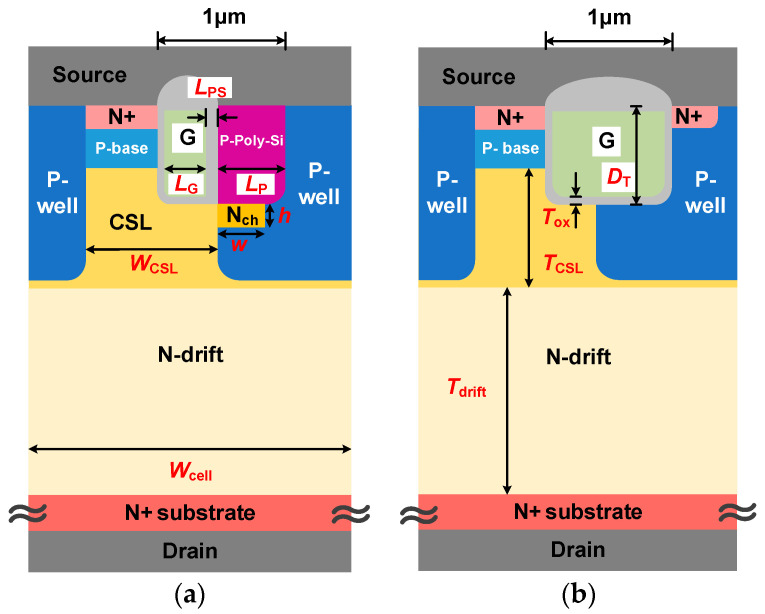
Schematic cross section of (**a**) HJD-ATMOS and (**b**) C-ATMOS.

The schematic cross section of HJD-ATMOS and C-ATMOS is shown in Figure 1. Similar to C-ATMOS, the device forms an inversion layer channel in the first quadrant to facilitate electron conduction. The N-CSL layer on the N-drift region reduces the on-resistance. Deep P-wells are used to reduce the electric field stress in the gate oxide at the trench bottom and corner [27]. The primary distinction is the body diode structure. The *N*_ch_ region under the P-Poly-Si provides a low-barrier path for electrons. Meanwhile, the HJD-ATMOS has a split gate and HJD structure on the right of the gate oxide layer. The split gate structure uses only a portion of the trench space for the gate electrode, while the other part is thicker P-Poly-Si that forms a portion of the HJD structure. The HJD-TMOS facilitates low-voltage conduction by allowing electrons to cross the lower heterojunction barrier in the third quadrant. The structure of *N*_ch_ and N-channel doping concentration (*N*_nch_) will be further discussed based on this optimization in this paper. Device specifications are presented in Table 1.

Sentaurus TCAD simulations are used to analyze the performances of the HJD-ATMOS and the C-ATMOS, considering doping and temperature-dependent Shockley–Read–Hall and Auger recombination, doping-dependent transport, impact ionization, band narrowing, high-field velocity saturation, and mobility degradation [29], as well as fixed charges at the SiC/SiO_2_ interface for closer simulation results to experimental data.

**Figure 2:**
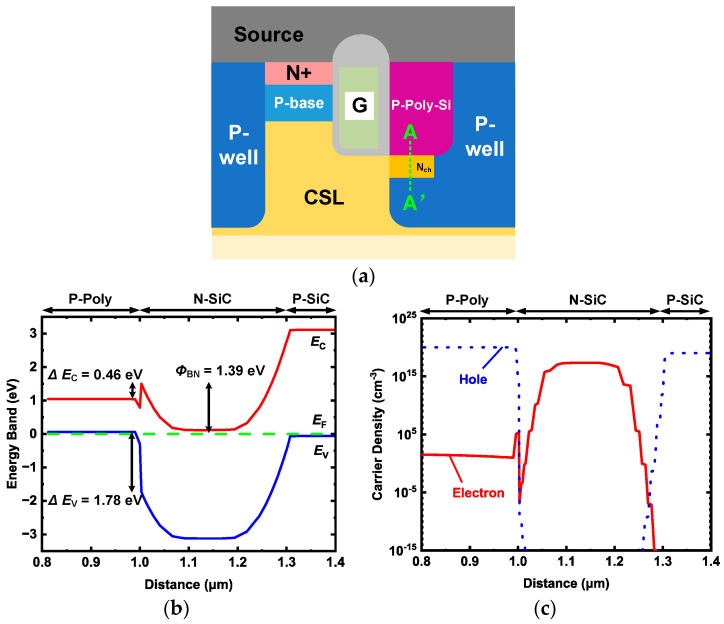
(**a**) Schematic cross section of HJD-ATMOS; (**b**) energy band diagram at thermal equilibrium along the A-A’ cut-line; (**c**) carrier density of the HJD when forward biased at rated voltage.

The energy band diagram of the P-Poly-Si/N-SiC heterojunction at thermal equilibrium is shown in Figure 2b. The energy band diagram at thermal equilibrium along the A-A’ cut-line is shown in Figure 2a. The heterojunction has a conduction energy gap of 0.46 eV and a valence barrier energy gap of 1.78 eV. The electron barrier height *Φ*_BN_ is determined by the Fermi level energy *E*_f_ and the conduction band peak energy *E*_c_, which is about 1.39 eV. Figure 2c shows the simulated carrier density at the heterojunction interface under forward bias at the rated voltage. Electrons are injected from N-SiC to P-poly, but there are few holes from P-poly to N-SiC due to the high hole barrier. Therefore, the HJD exhibits unipolar action, similar to the SBD [30].

**Figure 3:**
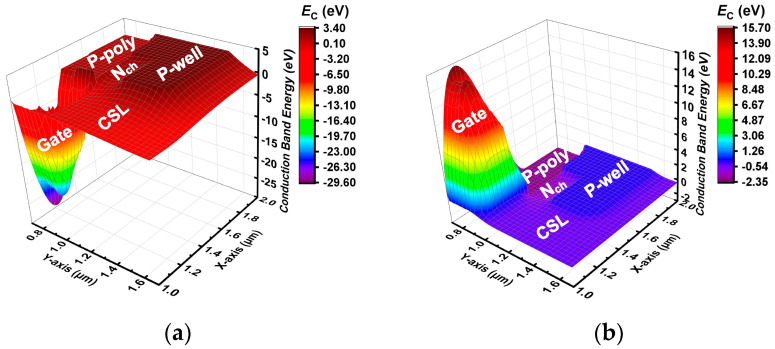
Three-dimensional conduction band energy distribution between P-poly, gate, *N*_ch_, N-CSL, and P-well (**a**) when conduction is forward and (**b**) when conduction is reverse.

We also constructed a 3D band diagram of the device to better observe the working state of the device. Figure 3a shows the 3-D conduction band energy distribution of the device at *V*_ds_ = 10 V and *V*_gs_ = 15 V. The band energy of *N*_ch_ is higher than that of N-CSL, which prevents electron current from flowing to P-poly and enables the device to work normally like C-ATMOS. Figure 3b shows the distribution of the devices when *V*_ds_ = −5 V and *V*_gs_ = −5 V. The band energy of *N*_ch_ is lower than that of N-CSL, resulting in electron current flowing from N-CSL to P-poly and preventing the turn-on of parasitic body-PN diodes.

**Figure 4:**
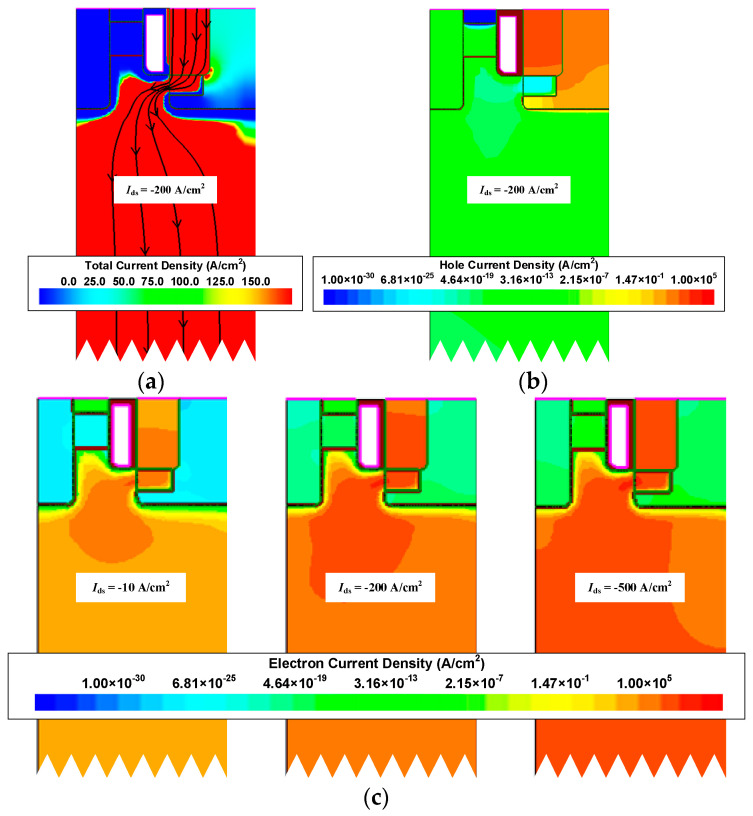
(**a**) Total current density distribution, (**b**) hole current density distribution, and (**c**) electron current density distribution at low and high current in the reverse conduction.

Figure 4 shows the distribution of the total current density, hole current density, and electron current density of the device. From the total current density distribution, it can be seen that the current does not flow from P-Poly-Si to P-well. But a high current density is also noted at the gate corner of P-Poly-Si, which should be noted in use. From the hole current density distribution, it can be seen that holes do not enter N-drift. This is due to the difference in the band gap between SiC and polysilicon. Since the energy barrier height between the SiC and polysilicon junctions in the valence band is very large, in the HJD-ATMOS, electron current can move toward the source while hole current cannot move toward the drain [31]. The device can operate normally at electron current densities of 10 A/cm^2^ and 500 A/cm^2^.

**Figure 5:**
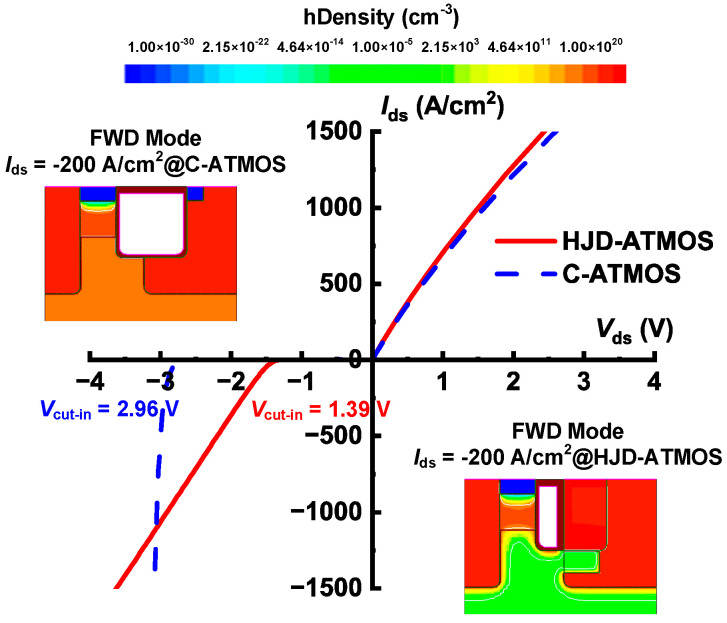
First and third quadrant characteristics of HJD-ATMOS and C-ATMOS.

Figure 5 shows the I–V curves of HJD-ATMOS and C-ATMOS in forward and reverse conduction at room temperature. The steeper slope of the I–V curve of HJD-ATMOS in the first quadrant indicates that its specific on-resistance (*R*_on,sp_) is lower than that of C-ATMOS. This is because the presence of *N*_ch_ in HJD-ATMOS results in a smaller depletion region of P-well on N-CSL, leading to a wider current conduction region. According to the calculations, at *V*_gs_ = 15 V and *I*_ds_ = 200 A/cm^2^, the *R*_on,sp_ values for HJD-ATMOS and C-ATMOS are 1.35 mΩ∙cm^2^ and 1.46 mΩ∙cm^2^, respectively. In the third quadrant, at *I*_ds_= −10 A/cm^2^, HJD-TMOS exhibits a significantly lower *V*_cut-in_ of only 1.39 V compared to the PN diode of C-TMOS. As a result, HJD-ATMOS is capable of reducing switching losses. The rated operating current of the device in the third quadrant is generally *I*_ds_ = −200 A/cm^2^ [8]. This means that the proposed HJD-ATMOS has a clear advantage over C-ATMOS in that it can start working at a lower voltage. The hole density distribution diagram in Figure 5 for *I*_ds_ = −200 A/cm^2^ shows that the integrated HJD effectively suppresses minority carrier injection, reducing bipolar degradation.

**Figure 6:**
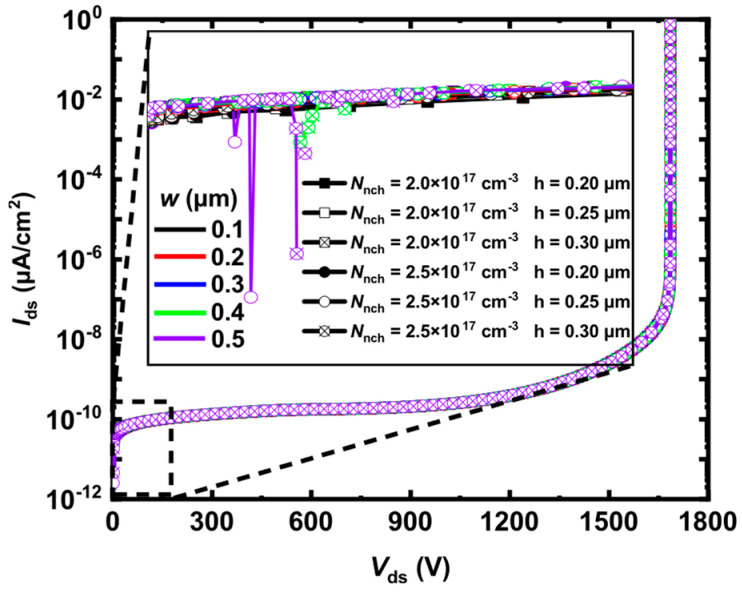
The breakdown voltage varies with *h*, *w*, and *N*_nch_, when *N*_nch_ is 2.0 × 10^17^ cm^−3^ and 2.5 × 10^17^ cm^−3^, *h* is 0.20 μm, 0.25 μm and 0.30 μm, and *w* is 0.1 μm to 0.5 μm, respectively.

**Figure 7:**
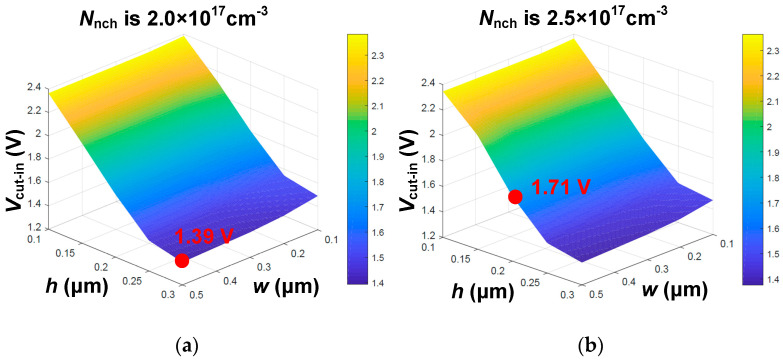
*V*_cut-in_ varies with *h*, *w*, and *N*_nch_, when *N*_nch_ is (**a**) 2.0 × 10^17^ cm^−3^, and (**b**) 2.5 × 10^17^ cm^−3^.

**Figure 8:**
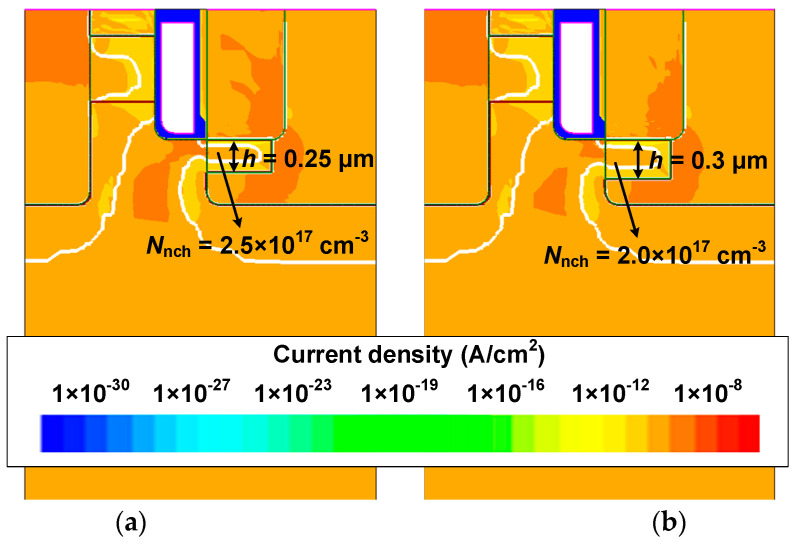
Current density distribution when (**a**) *N*_nch_ is 2.5 × 10^17^ cm^−3^, *h* is 0.25 μm, and (**b**) *N*_nch_ is 2.0 × 10^17^ cm^−3^, *h* is 0.30 μm at *V*_ds_ = 0 V.

In Figure 6, the local magnification shows that the HJD-ATMOS is affected by current spikes due to leakage. The figure demonstrates the change in breakdown voltage as a function of *h* and *w* when *N*_nch_ is, respectively, 2 × 10^17^ cm^−3^ and 2.5 × 10^17^ cm^−3^. It can be observed that when *N*_nch_ is 2.5 × 10^17^ cm^−3^, with *h* at 0.25 μm and *w* at 0.5 μm, the spike in the current is large, indicating the occurrence of leakage. When *N*_nch_ is 2.5 × 10^17^ cm^−3^, increasing *h* to 0.30 μm and *w* to 0.4 μm also results in leakage. However, when *N*_nch_ is 2.0 × 10^17^ cm^−3^ and *h* increases to 0.3 μm, the device does not exhibit leakage, demonstrating that variations in *N*_nch_ have a significant impact on device performance. As shown in Figure 7, *V*_cut-in_ varies significantly with *h*. The minimum point of *V*_cut-in_ is 1.31 V at *N*_nch_ = 2 × 10^17^ cm^−3^, which is lower compared to its value of 1.71 V at *N*_nch_ = 2.5 × 10^17^ cm^−3^ and *h* = 0.2 μm. This point represents the critical condition for the device not exhibiting leakage when *N*_nch_ = 2.5 × 10^17^ cm^−3^. After *h* is greater than 0.25 μm, the variation in *V*_cut-in_ with *h* tends to be flat, and if the value of *h* is larger, the protective effect of P-well on the gate oxide will also be weakened, and it will also increase the difficulty of process manufacturing. As can be seen from Figure 8, when the device *V*_ds_ is 0 V, *N*_nch_ is 2.5 × 10^17^ cm^−3^, and *h* is 0.25 μm, the HJD-ATMOS has more leakage than the device with *N*_nch_ is 2.0 × 10^17^ cm^−3^ and *h* is 0.30 μm. The darker regions in the current density plot for the HJD-ATMOS with *N*_nch_ at 2.5 × 10^17^ cm^−3^ and *h* at 0.25 μm are larger than those with *N*_nch_ at 2.0 × 10^17^ cm^−3^ and *h* at 0.30 μm, indicating higher leakage currents. This also confirms the hypothesis that the breakdown voltage spike is caused by heterojunction leakage. So the results indicate that *N*_nch_ = 2 × 10^17^ cm^−3^, *h* = 0.3 μm, and *w* = 0.5 μm are the optimal values.

## 3. Simulation Results and Discussion

**Figure 9:**
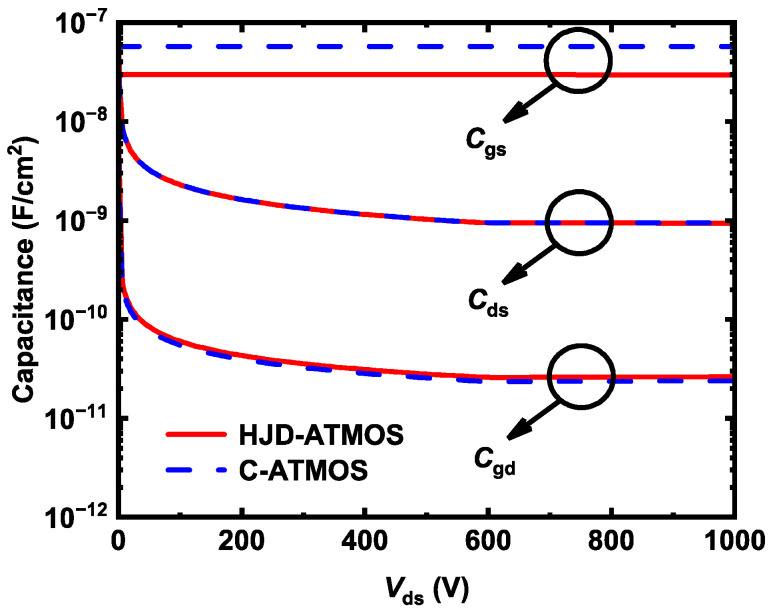
The device capacitance of HJD-ATMOS and C-ATMOS.

Figure 9 shows the capacitances of HJD-ATMOS and C-ATMOS. Gate voltage was fixed at 0 V, a 1 MHz AC signal was applied [32,33], and drain voltage was swept from 0 to 1000 V. HJD-ATMOS has lower gate-to-source capacitance (*C*_gs_) than C-ATMOS due to the smaller contact area with the source caused by the split gate structure. HJD-ATMOS’s gate-to-drain capacitance (*C*_gd_) does not decrease. This is because the P-well blocks the right side of the gate of C-ATMOS, performing a similar function as the split gate. Therefore, it can be observed that the *C*_iss_ (*C*_gs_ + *C*_gd_) of the HJD-ATMOS with split gates is also smaller than that of the C-ATMOS.

**Figure 10:**
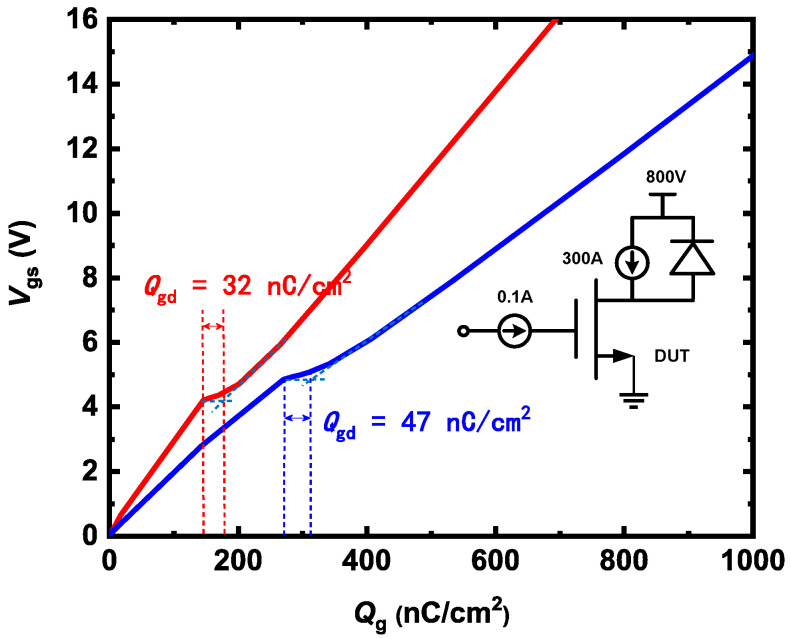
The gate charge characteristics of HJD-ATMOS and C-ATMOS.

Gate-to-drain charge (*Q*_gd_) is critical for power device switching speed in device applications. Figure 10 shows a test circuit to simulate HJD-ATMOS and C-ATMOS gate charges during turn-on. The miller plateau height of HJD-ATMOS is less than that of C-ATMOS, indicating that the threshold voltage of HJD-ATMOS is smaller than that of C-ATMOS [34]. Because the gate charge is proportional to the gate capacitance, the HJD-ATMOS has a lower gate charge (*Q*_g_) and *Q*_gd_ compared with the C-ATMOS. The Miller platform in HJD-ATMOS is shorter because of the reduced gate area. The *Q*_gd_ values for HJD-ATMOS and C-ATMOS are 32 nC/cm^2^ and 47 nC/cm^2^, respectively. *Q*_gd_ of HJD-ATMOS decreased by 31.91% compared to C-ATMOS. Reduced *Q*_gd_ leads to a smaller high-frequency figure of merit in HJD-ATMOS.

**Figure 11:**
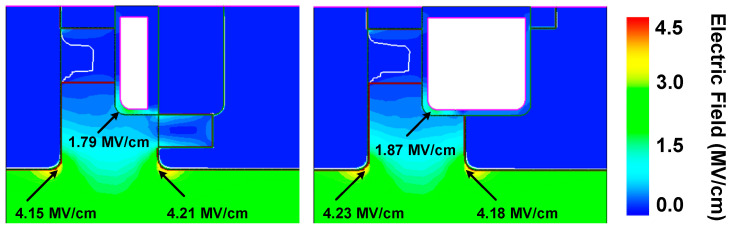
Electric field distribution for the HJD-ATMOS and the C-ATMOS at BV.

**Figure 12:**
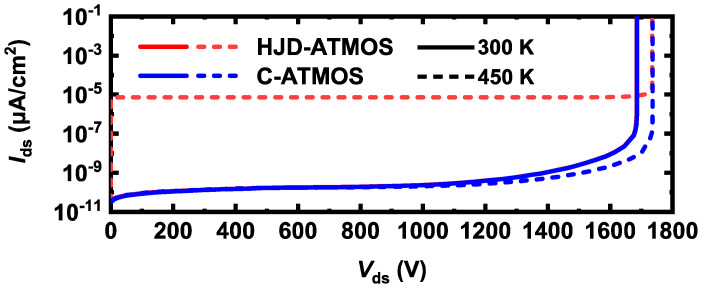
Blocking characteristics of the HJD-ATMOS and the C-ATMOS.

Figure 11 shows the electric field distribution at the breakdown of HJD-ATMOS and C-ATMOS. The electric field at the gate oxide of HJD-ATMOS is smaller than that of C-ATMOS. This is because the presence of the *N*_ch_ introduces a portion of the electric field into this region, which alleviates the electric field that the gate oxide withstands. Although increasing the electric field at the heterojunction raises leakage current risk, it is a trade-off for improved reverse conduction performance. Figure 12 shows the blocking characteristics of the HJD-ATMOS and the C-ATMOS at room temperature and high temperature. At room temperature, the data are represented by solid lines, whereas at elevated temperatures, they are depicted by dashed lines. HJD-ATMOS and C-ATMOS have similar breakdown voltages at room temperature. But the leakage current of the HJD-ATMOS increases at high temperature due to the increased thermal energy of the charge carriers. The generation of leakage currents, as demonstrated and discussed in Figure 6 and Figure 8, arises due to leakage occurring at the heterojunction, where higher *N*_nch_ and greater values of thickness *h* both contribute to this effect. By improving the semiconductor material growth process, reducing defects and traps, and enhancing the material quality and interface integrity, it is possible to mitigate non-ideal scattering and trap effects experienced by charge carriers at the heterojunction interface, thus suppressing the leakage current. As discussed in reference [20,35,36], regarding leakage current, while the HJD-ATMOS structure does indeed experience leakage under temperature influence, this leakage is within acceptable limits, with the level of leakage current being 1 × 10^−5^ μA/cm^2^.

**Figure 13:**
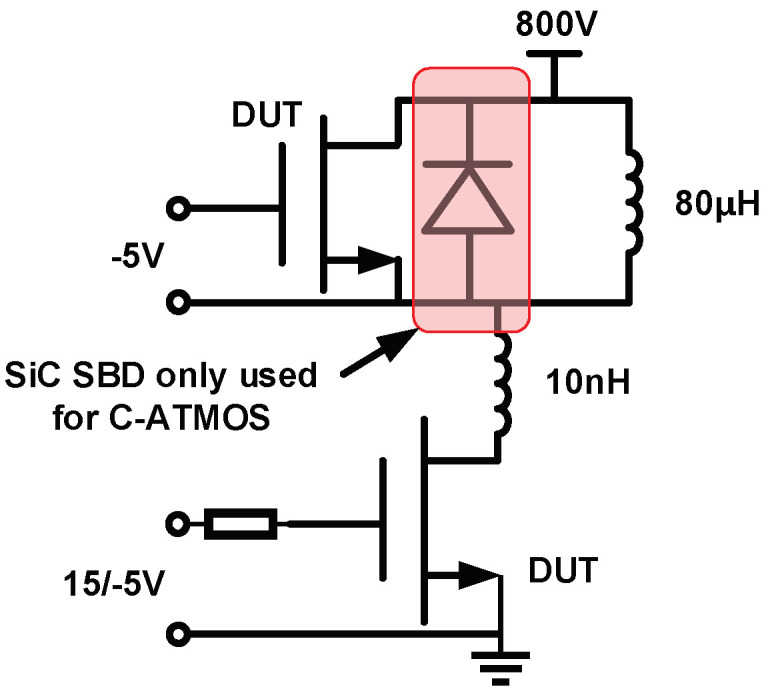
A circuit for simulating switching with a double pulsed test.

**Figure 14:**
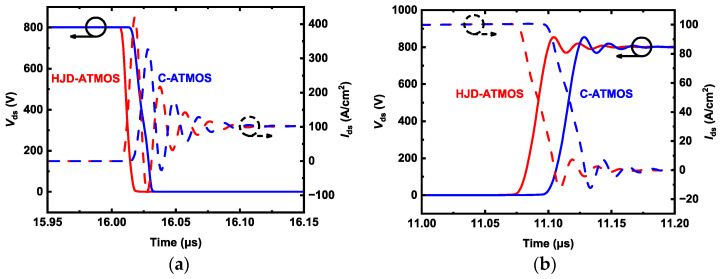
The switching characteristics of HJD-ATMOS and C-ATMOS, including the (**a**) turn-on process and (**b**) turn-off process.

**Figure 15:**
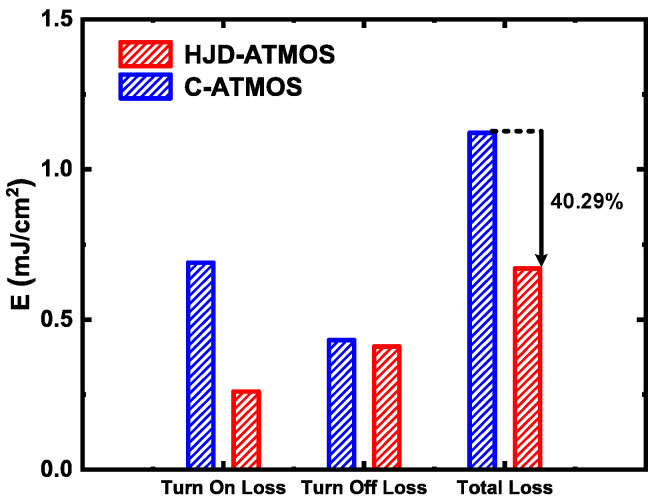
Switching loss comparison of IJ-ATMOS and C-ATMOS.

Figure 13 shows a double pulse test circuit for investigating switching characteristics. This is a common circuit configuration employed in device testing [16]. Stray inductance is 10 nH, and load inductance is 80 μH. The gate voltage source (*V*_g_) is turned on from −5 V to 15 V at t = 16 µs and turned off from 15 V to 0 V at t = 11 µs. Figure 14 shows the switching waveforms of devices. The switching speed of the HJD-ATMOS is faster than that of the C-ATMOS with an external SBD diode, which results in a smaller switching loss. Figure 15 compares the switching losses between the two devices. In HJD-ATMOS, the turn-on loss (*E*_on_) is 0.26 mJ/cm^2^, and the turn-off loss (*E*_off_) is 0.41 mJ/cm^2^, which demonstrate a reduction of 62.32% and 4.65%, respectively, compared to C-ATMOS. The total switch loss of HJD-ATMOS is reduced by 40.29% compared to C-ATMOS. This is due to the smaller *Q*_gd_ compared with the C-ATMOS. Reduced switching losses in power electronic devices are instrumental in improving operational longevity and reliability. As losses during switching are directly proportional to heat generation, a significant decrease in these losses curtails thermal build-up, mitigating the risk of device overheating and extending its operational life. This reduction also sustains lower junction temperatures, crucial for preventing material degradation in high-power-density applications where maintaining low operating temperatures is vital for ensuring long-term stability and reliability. Furthermore, minimizing switching losses allows power converters and similar equipment to function efficiently at elevated frequencies without sacrificing efficiency, empowering designers to develop compact, lightweight systems while consistently meeting reliability standards.

The majority of the process steps for HJD-ATMOS, including epitaxial growth, N+ source and P-well implantation, trench etching, P-base implantation, isolation oxidation, gate oxidation, polysilicon gate deposition, and metallization, are fully compatible with the manufacturing processes of C-ATMOS. The N-channel region is formed by ion implantation at the bottom of the trench after trench etching [37]. The split gate is formed by etching after trench oxidation, resulting in a thin layer of oxide between the gate and the P-Poly-Si. The gate-P-Poly-Si trench isolation layer is formed by thermal oxidation, and the trench oxide layer is fully etched and filled with P-Poly-Si.

Table 2 compares the HJD-ATMOS and the C-ATMOS in terms of their main characteristics. Dynamic FOM indicates the value of *R*_on,sp_ × *Q*_gd_ [38]. The HJD-ATMOS performs better due to the integrated HJD structure.

## 4. Conclusions

This paper proposes a novel asymmetric trench SiC MOSFET with a heterojunction diode. The performance of HJD-ATMOS and C-ATMOS is compared in detail. It can be observed that HJD-ATMOS demonstrates superior third-quadrant performance with a lower *V*_cut-in_ because of the integrated HJD. Compared with C-ATMOS, the *Q*_gd_ of HJD-ATMOS has decreased by 31.91%. This is because the split gate design further reduces the total gate charge, which reduces the switching loss of the HJD-ATMOS device without affecting other key characteristics. As a result, HJD-ATMOS eliminates bipolar degradation and reduces the turn-on loss from 0.69 mJ/cm^2^ in C-ATMOS to 0.26 mJ/cm^2^. With its advantageous features, HJD-ATMOS is a strong contender for power electronic applications.

## Figures and Tables

**Table 1:** Main parameters used in the simulation.

Parameter	HJD-ATMOS	C-ATMOS
*T*_drift_	9 μm	9 μm
*T*_CSL_	1 μm	1 μm
*W*_CSL_	0.9 μm	0.9 μm
*N*_drift_	7 × 10^15^ cm^−3^	7 × 10^15^ cm^−3^
*W*_cell_	2.7 μm	2.7 μm
*T*_OX_	50 nm	50 nm
*D*_T_	1 μm	1 μm
*L*_G_	0.25 μm	-
*L*_P_	0.6 μm	-
*L*_GP_	0.1 μm	-
*h*	0.3 μm	-
*w*	0.5 μm	-
*N*_nch_	2 × 1017 cm^−3^	-
*N*_P-Poly-Si_	1 × 1020 cm^−3^	-
*N*_CSL_	2.5 × 10^16^ cm^−3^	2.5 × 10^16^ cm^−3^
Fixed charges (SiC/SiO_2_)	6 × 10^11^ cm^−2^	6 × 10^11^ cm^−2^ [28]

**Table 2:** Device characteristics comparison.

Parameter	HJD-ATMOS	C-ATMOS
*V*_cut-in_	1.39 V	2.96 V
*R*_on,sp_	1.35 mΩ∙cm^2^	1.46 mΩ∙cm^2^
*Q*_gd_	32 nC/cm^2^	47 nC/cm^2^
BV	1685.39 V	1686.21 V
*E*_on_	0.26 mJ/cm^2^	0.69 mJ/cm^2^
*E*_off_	0.41 mJ/cm^2^	0.43 mJ/cm^2^
*V*_th_	4.28 V	4.86 V
Dynamic FOM	43.20 mΩ∙nC	68.62 mΩ∙nC

## Data Availability

Data is contained within the article.

## References

[1] Vathulya V.R., Shang H., White M.H. (1999). A novel 6H-SiC power DMOSFET with implanted p-well spacer. IEEE Electron Device Lett..

[2] Cabello M., Soler V., Rius G., Montserrat J., Rebollo J., Godignon P. (2018). Advanced processing for mobility improvement in 4H-SiC MOSFETs: A review. Mater. Sci. Semicond. Process..

[3] Huang R., Tao Y., Bai S., Chen G., Wang L., Liu A., Wei N., Li Y., Zhao Z. (2015). Design and fabrication of a 3.3 kV 4H-SiC MOSFET. J. Semicond..

[4] Meli A., Muoio A., Reitano R., Sangregorio E., Calcagno L., Trotta A., Parisi M., Meda L., La Via F. (2022). Effect of the Oxidation Process on Carrier Lifetime and on SF Defects of 4H SiC Thick Epilayer for Detection Applications. Micromachines.

[5] Lee G., Ha J., Kim K., Bae H., Kim C.-E., Kim J. (2022). Influence of Radiation-Induced Displacement Defect in 1.2 kV SiC Metal-Oxide-Semiconductor Field-Effect Transistors. Micromachines.

[6] Li J., Cheng X., Wang Q., Zheng L., Shen L., Li X., Zhang D., Zhu H., Shen D., Yu Y. (2017). Morphology improvement of SiC trench by inductively coupled plasma etching using Ni/Al_2_O_3_ bilayer mask. Mater. Sci. Semicond. Process..

[7] Liu G., Tuttle B.R., Dhar S. (2015). Silicon carbide: A unique platform for metal-oxide-semiconductor physics. Appl. Phys. Rev..

[8] Zhou C.Y., Ren M., Li X., Ma R.Y., Zhang X., Zheng F., Liang S.Q., Li Z.H., Zhang B. 4H-SiC Trench MOSFET with Integrated Heterojunction Diode for Optimizing Switching Performance. Proceedings of the 2022 IEEE 16th International Conference on Solid-State & Integrated Circuit Technology (ICSICT).

[9] Guo J., Li P., Ma R., Hu S. A Novel Asymmetric Trench SiC MOSFET Embedded Unipolar Electron Channel with Improved Reverse Conduction Performance. Proceedings of the 2022 IEEE 16th International Conference on Solid-State & Integrated Circuit Technology (ICSICT).

[10] Agarwal A., Fatima H., Haney S., Ryu S.H. (2007). A New Degradation Mechanism in High-Voltage SiC Power MOSFETs. IEEE Electron Device Lett..

[11] Palanisamy S., Basler T., Lutz J., Künzel C., Wehrhahn-Kilian L., Elpelt R. Investigation of the bipolar degradation of SiC MOSFET body diodes and the influence of current density. Proceedings of the 2021 IEEE International Reliability Physics Symposium (IRPS).

[12] Carastro F., Mari J., Zoels T., Rowden B., Losee P., Stevanovic L. Investigation on diode surge forward current ruggedness of Si and SiC power modules. Proceedings of the 2016 18th European Conference on Power Electronics and Applications (EPE’16 ECCE Europe).

[13] She X., Huang A.Q., Lucía Ó., Ozpineci B. (2017). Review of Silicon Carbide Power Devices and Their Applications. IEEE Trans. Ind. Electron..

[14] Sung W., Baliga B.J. (2016). Monolithically Integrated 4H-SiC MOSFET and JBS Diode (JBSFET) Using a Single Ohmic/Schottky Process Scheme. IEEE Electron Device Lett..

[15] Sung W., Baliga B.J. (2017). On Developing One-Chip Integration of 1.2 kV SiC MOSFET and JBS Diode (JBSFET). IEEE Trans. Ind. Electron..

[16] Zhang J., Chen Z., Tu Y., Deng X., Zhang B. (2021). A Novel SiC Asymmetric Cell Trench MOSFET with Split Gate and Integrated JBS Diode. IEEE J. Electron Devices Soc..

[17] Yu H., Wang J., Deng G., Liang S., Liu H., Shen Z.J. (2022). A Novel 4H-SiC JBS-Integrated MOSFET with Self-Pinching Structure for Improved Short-Circuit Capability. IEEE Trans. Electron Devices.

[18] An J., Hu S. (2019). Heterojunction Diode Shielded SiC Split-Gate Trench MOSFET with Optimized Reverse Recovery Characteristic and Low Switching Loss. IEEE Access.

[19] Deng X., Xu X., Li X., Li X., Wen Y., Chen W. (2020). A Novel SiC MOSFET Embedding Low Barrier Diode with Enhanced Third Quadrant and Switching Performance. IEEE Electron Device Lett..

[20] Yu H., Liang S., Liu H., Wang J., Shen Z.J. (2021). Numerical Study of SiC MOSFET with Integrated n-/n-Type Poly-Si/SiC Heterojunction Freewheeling Diode. IEEE Trans. Electron Devices.

[21] Shenoy P.M., Baliga B.J. (1997). High voltage P^+^ polysilicon/N^−^ 6H-SiC heterojunction diodes. Electron. Lett..

[22] Yamagami S., Hayashi T., Hoshi M. (2012). Novel Low V_ON_ Poly-Si/4H-SiC Heterojunction Diode Using Energy Barrier Height Control. Mater. Sci. Forum.

[23] Ni W., Emori K., Marui T., Saito Y., Yamagami S., Hayashi T., Hoshi M. (2014). SiC Trench MOSFET with an Integrated Low Von Unipolar Heterojunction Diode. Mater. Sci. Forum.

[24] Fu H., Wei Z., Liu S., Wei J., Xu H., Ni L., Yang Z., Sun W. (2021). 1200V 4H-SiC trench MOSFET with superior figure of merit and suppressed quasi-saturation effect. Microelectron. Reliab..

[25] Siemieniec R., Peters D., Esteve R., Bergner W., Kück D., Aichinger T., Basler T., Zippelius B. A SiC Trench MOSFET concept offering improved channel mobility and high reliability. Proceedings of the 2017 19th European Conference on Power Electronics and Applications (EPE’17 ECCE Europe).

[26] Peters D., Basler T., Zippelius B., Aichinger T., Bergner W., Esteve R., Kueck D., Siemieniec R. The New CoolSiC™ Trench MOSFET Technology for Low Gate Oxide Stress and High Performance. Proceedings of the PCIM Europe 2017; International Exhibition and Conference for Power Electronics, Intelligent Motion, Renewable Energy and Energy Management.

[27] Peters D., Siemieniec R., Aichinger T., Basler T., Esteve R., Bergner W., Kueck D. Performance and ruggedness of 1200V SiC—Trench—MOSFET. Proceedings of the 2017 29th International Symposium on Power Semiconductor Devices and IC’s (ISPSD).

[28] Afanasev V.V., Bassler M., Pensl G., Schulz M. (1997). Intrinsic SiC/SiO_2_ Interface States. Phys. Status Solidi (A).

[29] (2016). TCAD Sentaurus Device Manual.

[30] Tanaka H., Hayashi T., Shimoida Y., Yamagami S., Tanimoto S., Hoshi M. Ultra-low Von and High Voltage 4H-SiC Heterojunction Diode. Proceedings of the Proceedings. ISPSD ‘05. The 17th International Symposium on Power Semiconductor Devices and ICs.

[31] Na J., Cheon J., Kim K. (2021). 4H-SiC Double Trench MOSFET with Split Heterojunction Gate for Improving Switching Characteristics. Materials.

[32] Zhang M., Wei J., Jiang H., Chen K.J., Cheng C.H. (2017). A New SiC Trench MOSFET Structure with Protruded p-Base for Low Oxide Field and Enhanced Switching Performance. IEEE Trans. Device Mater. Reliab..

[33] Wei J., Zhang M., Jiang H., Wang H., Chen K.J. (2017). Dynamic Degradation in SiC Trench MOSFET with a Floating p-Shield Revealed with Numerical Simulations. IEEE Trans. Electron Devices.

[34] Xu H.Y., Wang Y., Bao M.T., Cao F. (2022). Low Switching Loss Split-Gate 4H-SiC MOSFET with Integrated Heterojunction Diode. IEEE J. Electron Devices Soc..

[35] Na J., Kim K. 3.3 kV 4H-SiC MOSFET with embeded hetero junction body diode for low switching loss. Proceedings of the 2022 International Conference on Electronics, Information, and Communication (ICEIC).

[36] Yu Y., Liu T., Ma R., Cheng Z., Tao J., Guo J., Wu H., Hu S. (2024). A Novel Asymmetric Trench SiC MOSFET with an Integrated JFET for Improved Reverse Conduction Performance. IEEE Trans. Electron Devices.

[37] Ding J., Deng X., Li S., Wu H., Li X., Li X., Chen W., Zhang B. (2022). A Low-Loss Diode Integrated SiC Trench MOSFET for Improving Switching Performance. IEEE Trans. Electron Devices.

[38] Yang T., Wang Y., Yue R. (2020). A heterojunction-based SiC power double trench MOSFET with improved switching performance and reverse recovery. Superlattices Microstruct..

